# An interpretable machine learning model for predicting response to anti-GD2 combined with umbilical cord blood-derived natural killer cell immunotherapy in neuroblastoma

**DOI:** 10.3389/fimmu.2026.1940534

**Published:** 2026-10-29

**Authors:** Qiqing Dong, Mengjia Song, Yumiao Mai, Juan Wang, Jia Zhu, Suying Lu, Feifei Sun, Junting Huang, Zijun Zhen, Xiya Tan, Yu Zhang, Yi Que, Yingxia Lan, Yongjun Cao, Fuqin Deng, Hao Qin, Yizhuo Zhang, Tong Xiang

**Affiliations:** 1 School of Electronics and Information Engineering, Wuyi University, Jiangmen, China; 2 State Key Laboratory of Oncology in South China, Guangdong Provincial Clinical Research Center for Cancer, Collaborative Innovation Center for Cancer Medicine, Department of Pediatric Oncology, Sun Yat-sen University Cancer Center, Guangzhou, China; 3 State Key Laboratory of Oncology in South China, Guangdong Provincial Clinical Research Center for Cancer, Collaborative Innovation Center for Cancer Medicine, Department of Anesthesiology, Sun Yat-sen University Cancer Center, Guangzhou, China; 4 Institute of Intelligent Manufacturing, Guangdong Academy of Sciences, Guangzhou, China; 5 State Key Laboratory of Oncology in South China, Guangdong Provincial Clinical Research Center for Cancer, Collaborative Innovation Center for Cancer Medicine, Department of Experimental Research, Sun Yat-sen University Cancer Center, Guangzhou, China; 6 NMPA Center for Innovation and Research in Regulatory Science, Guangzhou, China

**Keywords:** machine learning, neuroblastoma, peripheral blood testing, response prediction, umbilical cord blood-derived natural killer cell immunotherapy

## Abstract

**Background:**

Neuroblastoma remains a major therapeutic challenge for children with high-risk or relapsed/refractory disease. Anti-GD2 therapy combined with umbilical cord blood-derived natural killer (UCB-NK) cell therapy represents an emerging strategy, but responses vary substantially. Imaging and bone marrow assessments require specialized resources, creating a need for complementary tools based on routinely available data. However, whether serial routine peripheral blood measurements can support response prediction in this treatment setting remains unclear.

**Methods:**

Serial peripheral blood measurements from 42 patients were used to derive dynamic coupling features that reflected longitudinal trends and coordinated changes between biomarkers. These features were integrated with static variables to develop machine learning models for response prediction. Seven machine learning models were compared using the same repeated stratified cross-validation framework. Model selection considered discrimination, probability-estimation quality, and interpretability. SHapley Additive exPlanations (SHAP) was used to interpret the final model.

**Results:**

Considering cross-validated performance, the quality of probability estimates, and clinical interpretability, L2-regularized logistic regression was selected as the final model for response prediction. The model leveraged dynamic peripheral blood features to predict response to anti-GD2 combined with UCB-NK cell immunotherapy, achieving a cross-validated AUC of 0.873 (95% bootstrap CI, 0.735–0.976) and a Brier score of 0.131 (95% bootstrap CI, 0.087–0.182). SHAP analysis characterized the direction and magnitude of feature contributions to model predictions.

**Conclusion:**

This study developed an interpretable response-prediction model using serial routine peripheral blood tests obtained in clinical practice. The model may complement treatment-response assessment using routinely available data. However, validation in larger, independent cohorts is required before clinical use.

## 1 Introduction

Neuroblastoma is a highly malignant solid tumor arising from embryonic neural crest cells and is among the most common extracranial solid tumors in infants and young children. It exhibits marked clinical and biological heterogeneity. Neuroblastoma accounts for approximately 8%–10% of all childhood malignancies and is responsible for about 15% of pediatric cancer-related deaths. It is also associated with a high risk of relapse and an unfavorable prognosis (1). According to the International Neuroblastoma Risk Group (INRG) stratification system, the long-term overall survival of patients with high-risk disease remains below 50% despite intensive multimodal therapy, including surgery, multi-agent chemotherapy, radiotherapy, and autologous hematopoietic stem cell transplantation (2). Outcomes are even poorer in patients with relapsed or refractory neuroblastoma. Approximately 20% of patients with high-risk neuroblastoma have refractory disease after standard induction therapy and nearly 50% relapse after achieving an initial remission (3). Improved treatment-response stratification therefore remains an important clinical need for patients with high-risk or relapsed/refractory neuroblastoma.

Cellular immunotherapy is being investigated for high-risk or relapsed/refractory neuroblastoma because of its potential to eliminate tumor cells and address treatment resistance (4–7). Among these strategies, natural killer (NK) cells are a leading effector-cell platform. They can kill tumor cells without prior antigen sensitization and can enhance antibody-mediated antitumor activity through antibody-dependent cellular cytotoxicity (ADCC) (8–10). Anti-GD2 therapy partly relies on NK cell-mediated ADCC; however, the efficacy of this mechanism may be constrained by treatment-associated NK cell exhaustion and impaired effector cell fitness (11). Previous studies have explored haploidentical or autologous NK cell-based immunotherapies in combination with anti-GD2 antibodies for neuroblastoma (12–14). Haploidentical NK cells may be limited by human leukocyte antigen (HLA)-related rejection and insufficient expansion or persistence, whereas autologous NK cells may be functionally compromised by prior therapy, resulting in suboptimal effector activity (15). Umbilical cord blood–derived natural killer (UCB-NK) cells are particularly attractive as an allogeneic and potentially off-the-shelf effector-cell source. They are relatively accessible, exhibit robust ex vivo expansion capacity, have a favorable safety profile, and may offer functional advantages over NK cells isolated from patients’ peripheral blood. When combined with anti-GD2 therapy, UCB-NK cells are designed to augment NK cell–mediated ADCC. This strategy has demonstrated enhanced antitumor activity in neuroblastoma (16–18).

As anti-GD2 therapy combined with UCB-NK cell therapy enters early clinical translation, heterogeneous responses complicate individualized monitoring during treatment. Current neuroblastoma risk stratification and disease assessment use complementary approaches, including MYCN status, MIBG imaging, bone marrow assessments (19), and genomic analyses (20, 21). These approaches provide information on tumor biology or disease burden and support clinical evaluation. Their relevance to response prediction for anti-GD2 therapy combined with UCB-NK cell therapy requires separate evaluation. Some assessments require specialized platforms and institutional resources, which may limit their accessibility across centers (22). By contrast, peripheral blood testing is inexpensive and widely available (23–25). However, summaries of individual biomarker levels do not explicitly capture coordinated changes between biomarkers over time. In neuroblastoma and related immunotherapy settings, dynamic biomarker monitoring across multiple time points may capture disease status and treatment response more comprehensively than single baseline measurements (26). Together, these findings provide a rationale for evaluating serial peripheral blood data as an accessible source of complementary information for treatment-response prediction.

Whether coordinated changes in peripheral blood biomarkers can inform response prediction in this treatment setting remains unclear. To address this question, we developed an interpretable patient-level model integrating static biomarker summaries with dynamic coupling features derived from serial pre-infusion blood measurements. We compared predictive performance across models and examined feature contributions to predictions from the selected model. This exploratory study evaluated the approach as a potential complement to standard response assessment.

## 2 Materials and methods

This study was a retrospective cohort analysis based on a single-center phase I clinical trial conducted at Sun Yat-sen University Cancer Center, Guangzhou, China. The trial was registered at ClinicalTrials.gov (Registration No. NCT06631391). The study protocol was approved by the Ethics Committee of Sun Yat-sen University Cancer Center (Approval No. SL-B2024-008-02). Written informed consent was obtained from all patients or their legal guardians before enrollment.

### 2.1 Study cohort and data source

This retrospective cohort study included 61 pediatric patients with high-risk or relapsed/refractory neuroblastoma who participated in a phase I clinical study at Sun Yat-sen University Cancer Center between December 2024 and December 2025. Eligible patients had a histologic diagnosis, received anti-GD2 combined with UCB-NK cell immunotherapy, and had available serial blood measurements and response assessments. Patients were excluded for failure to receive the prescribed regimen, early death, loss to follow-up, or study withdrawal. This exploratory retrospective analysis included all eligible patients from the phase I clinical study during the specified study period. The sample size was determined by the number of eligible patients available. No formal sample size calculation was performed specifically for prediction model development.

According to the treatment strategy used in this phase I study, newly diagnosed high-risk patients received anti-GD2 combined with UCB-NK cell immunotherapy. For patients with relapsed or refractory disease, treatment was assigned according to clinical assessment. Patients with measurable or active disease requiring cytoreduction received chemotherapy combined with anti-GD2 and UCB-NK cell immunotherapy, whereas those without active lesions requiring chemotherapy received anti-GD2 combined with UCB-NK cell immunotherapy alone. These treatment-defined subgroups were used in exploratory subgroup analyses.

### 2.2 Study endpoints

The primary endpoint was binary treatment response, categorized as response or non-response. Models were developed to predict this endpoint from peripheral blood features (**Figure 1A**). Response was assessed according to the Response Evaluation Criteria in Solid Tumors, version 1.1 (RECIST v1.1) (27). Patients without measurable disease at baseline were classified as having either sustained complete response (SCR) or progressive disease (PD), whereas those with measurable disease at baseline were classified as having complete response (CR), partial response (PR), stable disease (SD), or progressive disease (PD).

**Figure 1:**
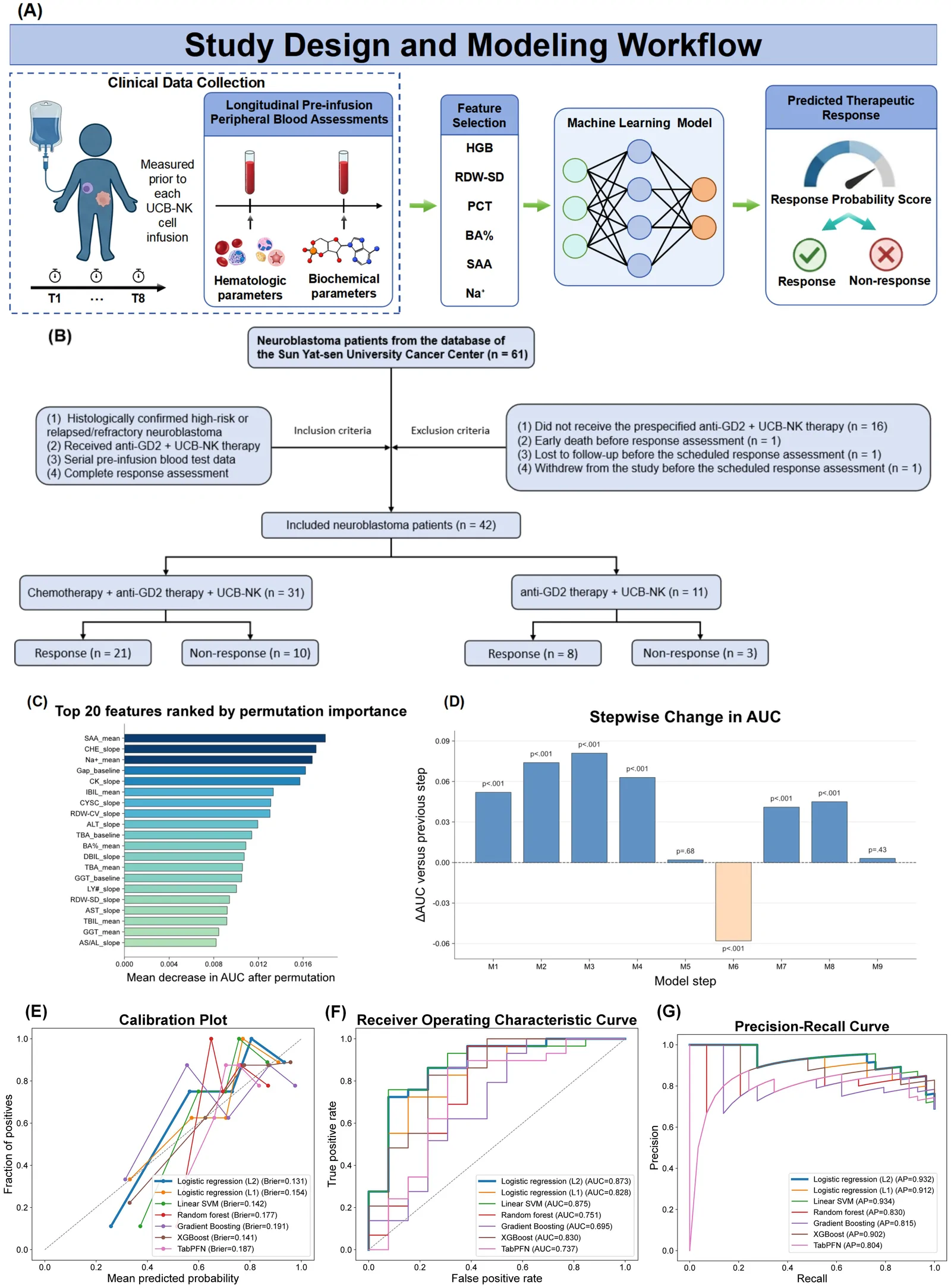
Study design, patient flow, feature selection, and model evaluation. **(A)** Schematic overview of the study design and modeling workflow. **(B)** Patient selection and composition of the analytical cohort. **(C)** Top 20 candidate features ranked by permutation importance. **(D)** Stepwise change in AUC during sequential feature addition. M0 is not shown because no preceding model was available for comparison. **(E)** Calibration curves. **(F)** Receiver operating characteristic curves. **(G)** Precision-recall (PR) curves. All candidate models were evaluated using the same repeated stratified cross-validation framework.

For the binary endpoint defined in this study, SCR, CR, and PR were classified as response, and SD and PD as non-response. In patients with measurable disease, SD did not meet the criteria for CR or PR. This grouping reflects response rather than disease control and does not equate SD with progression or lack of clinical benefit.

### 2.3 Selection of predictor variables

Peripheral blood measurements were collected before each of the eight UCB-NK cell infusions. They included 24 complete blood count parameters and 37 biochemical parameters, yielding 61 candidate variables. To account for within-patient dependence, repeated numeric variables were summarized at the patient level using the baseline value, mean, and Theil-Sen slope, representing initial status, overall level, and temporal trend, respectively. Baseline values were defined as measurements obtained before the first UCB-NK cell infusion. Patient-level means were calculated from available non-missing measurements within the observation period.

Exploratory univariate analyses examined associations between patient-level summary features and treatment response. Dynamic features were then constructed to characterize temporal trends and coordinated changes between biomarkers.

To identify dynamic coupling features, we performed within-patient analyses after centering measurements within each patient to reduce inter-individual baseline differences. Given the limited sample size, and to avoid deriving unstable dynamic associations driven by a few extreme individuals, pre-specified criteria for feature inclusion were established: (a) each group was required to include at least 10 patients; (b) the strength of within-patient coordinated associations was required to be ≥0.6 within the responder group; (c) the difference in association strength between the two groups was required to be ≥0.3. These criteria were intended to identify associations that were more stable in responders and weaker in non-responders.

Within each patient, observations missing either measurement were excluded for each biomarker pair retained after dynamic screening. Duplicate measurements at the same time point were averaged, and the remaining paired observations were ordered chronologically before feature calculation. Coupling slopes were estimated using Theil-Sen regression of Biomarker_B on Biomarker_A. Concordance rates were calculated as the proportion of eligible intervals between consecutive retained time points in which both biomarkers changed in the same direction. Intervals with zero change in either biomarker were excluded from the concordance calculation. Each coupling feature was recorded as missing when fewer than two paired time points remained or the corresponding statistic was undefined.

Regularized logistic regression was used to evaluate candidate feature sets. Feature screening was restricted to complete cases to avoid constructing longitudinal associations from imputed measurements. Candidate features were augmented by incorporating potential biomarkers identified in prior studies, based on dynamic feature screening results and clinical interpretability. To further identify variables that could contribute additional predictive gain, permutation importance analysis was performed on all candidate variables. For each cross-validation fold, the value of a single feature was randomly permuted while keeping all other features unchanged. The resulting decrease in AUC was used to quantify the loss of discriminative performance. This process was repeated 50 times to aggregate the importance distribution and assess its stability. Candidate feature combinations and model configurations were compared using repeated stratified cross-validation. The selected feature set was then used for model comparison in all 42 patients, with median imputation performed within training folds.

### 2.4 Model development and comparison

We compared L1- and L2-regularized logistic regression as interpretable baselines and a linear support vector machine (Linear SVM) as a margin-based alternative. Random forest, Gradient boosting, and XGBoost were included to model nonlinear relationships and interactions, while Tabular Prior-Data Fitted Network (TabPFN) provided a pretrained tabular comparator. All models were developed using the same set of features. The feature set was fixed before model comparison and was not reselected within cross-validation folds. Model development used patient-level, stratified 3-fold cross-validation repeated 50 times. Stratification by response status maintained class proportions comparable to those of the overall cohort in the training sets and held-out folds. Given the small cohort, model hyperparameters were not tuned during the comparative analysis. Instead, fixed model configurations were compared using the same cross-validation partitions defined at the patient level to reduce optimism introduced by tuning. For logistic regression with L1 or L2 regularization and for the Linear SVM, features were standardized using means and standard deviations estimated from each training fold. No external standardization was applied to models based on decision trees because their splitting procedures do not generally require feature scaling. TabPFN was also used without external standardization because it incorporates its own preprocessing. Linear SVM scores were converted to probabilities using sigmoid calibration with 3-fold internal cross-validation. Full model configurations and hyperparameter settings are reported in **Supplementary Table 1**. We also performed exploratory subgroup analyses by treatment regimen to assess the stability of model performance across therapeutic contexts.

### 2.5 Model performance evaluation

To evaluate the predictive performance of each model, we assessed discriminative ability using the area under the receiver operating characteristic curve (AUC) and the average precision (AP) score, which measures the model’s ability to identify responders. The quality of predicted probabilities was assessed using the Brier score and logarithmic loss (Log loss). Primary performance metrics were calculated from each patient’s out-of-fold response probability averaged across the 50 cross-validation repeats, using identical partitions across models. The classification threshold was selected by maximizing the Youden index (J = sensitivity + specificity − 1) (28). Patients were classified as responders if their predicted probabilities met or exceeded the selected threshold and as non-responders otherwise. Exploratory decision curve analysis (DCA) evaluated a hypothetical non-response alert using patient-level mean out-of-fold non-response probabilities (**Supplementary Methods**).

### 2.6 Model explanation

To improve transparency and clinical interpretability, we used SHapley Additive exPlanations (SHAP) to explain the final model. Following model selection, the L2-regularized logistic regression model was refitted to the full analysis cohort using median imputation and standardization. SHAP values were calculated using LinearExplainer for this refitted model rather than for the fold-specific cross-validation models. For the individual waterfall plots, out-of-fold probabilities were used only to select illustrative patients. The SHAP analysis described the refitted model, whereas predictive performance was evaluated using out-of-fold predictions from repeated stratified cross-validation. SHAP values quantify the magnitude and direction of feature contributions to individual model outputs, supporting global summaries of feature importance and patient-level explanations.

### 2.7 Statistical analysis

All analyses were performed in Python 3.8. Associations between categorical baseline variables and treatment outcome were assessed using Fisher’s exact test or Pearson’s χ² test, as appropriate based on expected cell frequencies. To control the false discovery rate (FDR) arising from multiple comparisons, the Benjamini-Hochberg procedure was applied, with adjusted p values reported as q values. Univariate associations between static aggregated features and treatment outcome were evaluated using Spearman’s rank correlation, and corresponding p values were calculated. For the predefined sequence of stepwise feature expansion models, performance was estimated using repeated stratified k-fold cross-validation (3 folds × 50 repeats). Within each repeated split, the performance difference between successive models was calculated. The Wilcoxon signed-rank test was applied to the resulting distribution of paired differences to assess the consistency of performance gains from added features across splits. In exploratory subgroup analyses, AUC distributions derived from the same repeated stratified cross-validation procedure were compared between treatment subgroups using the Wilcoxon rank-sum test. Event-free survival (EFS), progression-free survival (PFS), and overall survival (OS) were estimated using the Kaplan-Meier method. Differences in survival between groups were assessed using the log-rank test. A p value <.05 was considered statistically significant, and a q value <.05 was considered significant after FDR correction.

## 3 Results

### 3.1 Baseline characteristics

According to the predefined inclusion and exclusion criteria, 42 patients with high-risk or relapsed/refractory neuroblastoma who received anti-GD2 combined with UCB-NK cell immunotherapy were included in this study (**Figure 1B**). As shown in **Table 1**, the median age at diagnosis was 4 years (range, 1–11 years). Most patients (81%, 34/42) were aged ≤5 years. Males accounted for 64% of the cohort (27/42). Most patients had INRG stage M disease (93%, 39/42), and 95% were classified as high risk (40/42). Among them, 31 received chemotherapy combined with anti-GD2 and UCB-NK cell immunotherapy, whereas 11 received anti-GD2 combined with UCB-NK cell immunotherapy. For the efficacy endpoint, 29 of 42 patients (69.0%) were classified as treatment responders and 13 (31.0%) as non-responders, corresponding to a responder-to-non-responder ratio of 2.23:1.

**Table 1:** Baseline clinical characteristics of patients with neuroblastoma who received anti-GD2 combined with UCB-NK cell immunotherapy at Sun Yat-sen University Cancer Center between December 2024 and December 2025.

Characteristic	Total (n = 42)	Response (n = 29)	Non-response (n = 13)	*p* value
Gender				
Male	27 (64)	19 (66)	8 (62)	*p* >.99
Female	15 (36)	10 (34)	5 (38)	
Age				
≤5	34 (81)	26 (90)	8 (62)	*p* = .08
>5	8 (19)	3 (10)	5 (38)	
Disease Status				
Newly diagnosed high-risk	15 (36)	13 (45)	2 (15)	*p* = .15
Relapsed	7 (17)	4 (14)	3 (23)	
Refractory	20 (48)	12 (41)	8 (62)	
INRG stage				
L1	1 (2)	1 (3)	0 (0)	*p* = .49
L2	2 (5)	2 (7)	0 (0)	
M	39 (93)	26 (90)	13 (100)	
Risk group				
Low	1 (2)	1 (3)	0 (0)	*p* = .63
Intermediate	1 (2)	1 (3)	0 (0)	
High	40 (95)	27 (93)	13 (100)	
MYCN				
Amplified	10 (24)	7 (24)	3 (23)	*p* = .67
Not amplified	27 (64)	18 (62)	9 (69)	
Gain	1 (2)	1 (3)	0 (0)	
Unknown	4 (10)	3 (10)	1 (8)	
Treatment Subgroup				
Chemotherapy + anti-GD2 + UCB-NK	31 (74)	21 (72)	10 (77)	*p* >.99
anti-GD2 + UCB-NK	11 (26)	8 (28)	3 (23)	

Data are presented as n (%). Percentages may not sum to 100% due to rounding. Response was defined as SCR, CR, or PR. Non-response was defined as SD or PD. INRG, International Neuroblastoma Risk Group. MYCN, MYCN gene status. Unknown indicates that the information was missing or not tested. In the treatment subgroup, Chemotherapy + anti-GD2 + UCB-NK indicates chemotherapy combined with anti-GD2 and UCB-NK cell immunotherapy, and anti-GD2 + UCB-NK indicates anti-GD2 combined with UCB-NK cell immunotherapy.

### 3.2 Selection of predictor variables

INRG stage, risk classification, and MYCN status were not included in this correlation analysis because these variables are typically determined by imaging evaluation and tumor tissue or molecular testing rather than routine laboratory measurements. Spearman’s rank correlation analyses showed no significant associations between static features and treatment response. After FDR correction, all q values remained >.05. These univariate analyses provided no clear evidence of associations between individual static blood features and treatment response in this cohort.

Dynamic co-variation between laboratory parameters during treatment is summarized in **Table 2**. Associations of white blood cell count (WBC) with absolute neutrophil count (NE#), absolute lymphocyte count (LY#), and absolute monocyte count (MO#) were considered redundant because WBC is mathematically related to the summed leukocyte subpopulations (29). Neutrophil percentage (NE%) and lymphocyte percentage (LY%) also showed redundant inverse correlation under the compositional constraint of the white blood cell differential (30). Red blood cell distribution width-standard deviation (RDW-SD) and mean corpuscular volume (MCV) both reflect erythrocyte volume characteristics. Their association was not interpreted as independent mechanistic coupling (31). By contrast, plateletcrit (PCT) and basophil percentage (BA%) showed a consistent within-patient association that was not attributable to a shared derivation relationship. We therefore constructed two dynamic features to characterize coordinated changes between PCT and BA%: the Theil-Sen slope of BA% relative to PCT and a concordance rate defined as the proportion of adjacent intervals in which PCT and BA% changed in the same direction.

**Table 2:** Analysis of dynamic associations between biomarkers.

Biomarker_A	Biomarker_B	ρ	SE	n
WBC	NE#	0.880	0.073	40
MCV	MCHC	-0.818	0.073	40
NE%	LY%	-0.759	0.074	39
PCT	BA%	0.698	0.077	39
WBC	LY#	0.636	0.072	41
NE#	LY%	-0.620	0.072	41
RDW-SD	MCV	0.607	0.072	42

WBC, white blood cell count; NE#, absolute neutrophil count; MCV, mean corpuscular volume; MCHC, mean corpuscular hemoglobin concentration; NE%, neutrophil percentage; LY%, lymphocyte percentage; PCT, plateletcrit; BA%, basophil percentage; LY#, absolute lymphocyte count; RDW-SD, red blood cell distribution width-standard deviation. ρ, Spearman’s rank correlation coefficient. SE, standard error. n, number of patients included in each paired analysis.

Hemoglobin (HGB) in peripheral blood has been associated with prognosis across tumor types and may influence immune cell function and physiological responses (32). Meanwhile, anemia is common in neuroblastoma (33). We therefore used the mean HGB level during treatment (HGB_mean) as a patient-level summary. In neuroblastoma and other malignancies, red blood cell distribution width (RDW) has been associated with disease progression and stage (34). Higher preoperative RDW-SD has also been associated with improved overall survival (35). The baseline value of RDW-SD (RDW-SD_baseline) was therefore included as a candidate feature for response prediction.

Permutation importance analysis further identified mean serum amyloid A (SAA_mean), cholinesterase slope (CHE_slope), and mean sodium (Na+_mean) as among the most informative candidates across repeated cross-validation (**Figure 1C**). We therefore added these variables to the expanded candidate set and assessed their incremental value in subsequent stepwise modeling.

### 3.3 Model development and comparison

The feature set was expanded stepwise from the core candidate variables, and performance gains between adjacent models were evaluated under the same repeated stratified cross-validation framework. The addition of Na+_mean and SAA_mean each resulted in significant performance improvement. By contrast, adding CHE_slope in models M6 and M9 did not materially improve AUC performance. Model performance approached a plateau at M8. After the addition of CHE_slope, the mean AUC increased by only 0.004, and the difference was not statistically significant (p = .43; **Figure 1D**; **Supplementary Table 2**). Therefore, M8 was selected as the final model based on predictive performance, stability of feature importance, and clinical availability. The final model included six features: the Theil-Sen slope of BA% relative to PCT (BA%–PCT slope), HGB_mean, RDW-SD_baseline, the proportion of adjacent time intervals in which BA% and PCT changed in the same direction (BA%–PCT concordance rate), Na+_mean, and SAA_mean.

After defining the final feature set, we compared the performance of several machine learning models under the same repeated stratified cross-validation framework (**Table 3**, **Figures 1E–G**). Linear models showed the strongest overall discrimination. The linear SVM achieved the highest discriminative performance (AUC = 0.875; AP = 0.934), whereas L2-regularized logistic regression showed comparable discrimination (AUC = 0.873; AP = 0.932). In contrast, L1-regularized logistic regression exhibited reduced performance (AUC = 0.828; AP = 0.912). Among the evaluated models, L2-regularized logistic regression had the lowest point estimates for Brier score (0.131) and Log loss (0.414). The corresponding values for Linear SVM were 0.142 and 0.451, respectively. The calibration curve for L2-regularized logistic regression was closely aligned with the ideal calibration line (**Figure 1E**). Tree-based models showed larger deviations from the ideal calibration line in some probability ranges. Exploratory DCA showed that neither L2-regularized logistic regression nor Linear SVM consistently achieved higher net benefit across thresholds (**Supplementary Figure 4**). On the basis of discrimination, the quality of probability estimates, and interpretability, we selected L2-regularized logistic regression as the final model.

**Table 3:** Comparison of model performance.

Model	AUC (95% CI)	AP (95% CI)	Brier score (95% CI)	Log loss (95% CI)
Logistic regression (L2)	0.873 (0.735-0.976)	0.932 (0.855-0.988)	0.131 (0.087-0.182)	0.414 (0.306-0.533)
Logistic regression (L1)	0.828 (0.682-0.950)	0.912 (0.833-0.976)	0.154 (0.111-0.200)	0.475 (0.370-0.585)
Linear SVM	0.875 (0.740-0.979)	0.934 (0.855-0.990)	0.142 (0.108-0.179)	0.451 (0.374-0.531)
Random Forest	0.751 (0.552-0.926)	0.830 (0.713-0.958)	0.177 (0.144-0.212)	0.540 (0.458-0.629)
Gradient boosting	0.695 (0.491-0.875)	0.815 (0.698-0.933)	0.191 (0.127-0.256)	0.626 (0.410-0.862)
XGBoost	0.830 (0.666-0.958)	0.902 (0.807-0.980)	0.141 (0.092-0.194)	0.451 (0.315-0.603)
TabPFN	0.737 (0.546-0.912)	0.804 (0.693-0.954)	0.187 (0.158-0.215)	0.561 (0.493-0.636)

AUC and AP summarize discriminative and precision–recall performance, respectively, whereas Brier score and Log loss indicate probability-estimation quality. The 95% confidence intervals (CIs) were derived from 2000 patient-level bootstrap resamples.

### 3.4 Exploratory subgroup performance analysis

The overall-cohort Youden threshold was 0.740, derived from cross-validation predictions. At this threshold, the model achieved a sensitivity of 0.724 (95% CI, 0.545–0.880) and a specificity of 0.923 (95% CI, 0.750–1.000) in the overall cohort (**Supplementary Figure 1**). The same overall-cohort Youden threshold was then applied, without re-optimization, in exploratory analyses of the two treatment subgroups. Both the 0.5 threshold and the Youden threshold (0.740) were applied to each subgroup. The subgroup results showed consistent trends with the overall cohort. However, in the anti-GD2 combined with UCB-NK subgroup, threshold-related metrics showed greater variability due to its smaller sample size (**Supplementary Figure 2**, **Supplementary Table 3**).

To assess the applicability of the final model across different treatment subgroups, we calculated the AUC values for each subgroup within the same repeated stratified cross-validation framework. The results showed that in the chemotherapy combined with anti-GD2 and UCB-NK cell immunotherapy subgroup, the model’s AUC distribution was stable (0.891 ± 0.037). In contrast, the AUC showed greater variability in the anti-GD2 combined with UCB-NK subgroup (0.709 ± 0.115), indicating that the smaller sample size in this subgroup led to higher variability in the prediction results (**Supplementary Figure 3**).

In the high-risk cohort, the model-predicted response group exhibited significantly better EFS compared to the model-predicted non-response group (p = .006). While the OS analysis showed favorable survival trends for both groups, the difference between them did not reach statistical significance (p = .32, **Figures 2A, B**). In the relapsed/refractory cohort, both PFS and OS demonstrated trends of stratification, but these differences did not achieve statistical significance with the current sample size (**Figures 2C, D**). Survival patterns were directionally similar when patients were grouped by predicted or observed response (**Figures 2E–H**).

**Figure 2:**
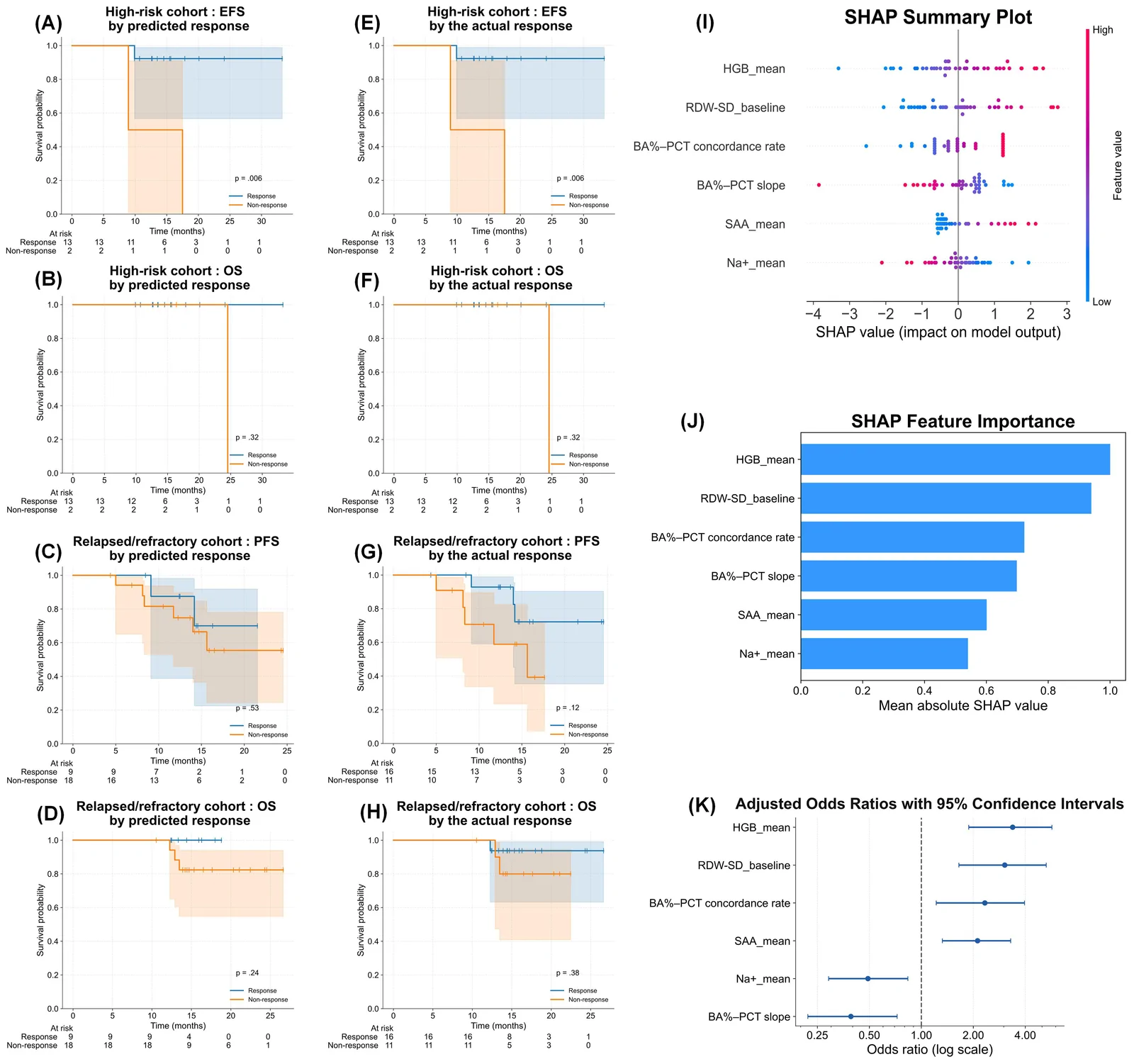
Kaplan-Meier survival curves and model interpretability analysis. **(A, B)** EFS and OS in the high-risk cohort by predicted response. **(C, D)** PFS and OS in the relapsed/refractory cohort by predicted response. **(E, F)** EFS and OS in the high-risk cohort by the actual treatment response. **(G, H)** PFS and OS in the relapsed/refractory cohort by the actual treatment response. **(I)** SHAP summary plot. **(J)** SHAP-based feature importance for the logistic regression model. SHAP values were calculated for the L2-regularized logistic regression model refitted to the full analysis cohort. **(K)** Forest plot of odds ratios (ORs). Dots represent point estimates; horizontal lines denote 95% confidence intervals (CIs). Each OR represents the effect of a 1-SD increase in the standardized predictor. CIs were estimated using 2000 bootstrap resamples.

### 3.5 Model explanation

We applied SHAP to interpret the final model. Higher values of HGB_mean, RDW-SD_baseline, the BA%–PCT concordance rate, and SAA_mean contributed positively to the prediction, while higher values of Na+_mean and the BA%–PCT slope contributed negatively. Mean absolute SHAP values quantified global contribution magnitude, while the beeswarm plot showed patient-level contribution directions and distributions (**Figures 2I, J**). **Figure 2K** shows that the overall direction of the coefficients was consistent with the SHAP analysis. The width of the confidence intervals reflects the statistical uncertainty of these estimates. Furthermore, dependence plots illustrate the relationship between each feature’s value and its SHAP value (**Supplementary Figures 5A–F**). A positive SHAP value shifts the prediction toward the responder class. Waterfall plots showed signed feature contributions to individual predicted response log-odds relative to the background expectation (**Supplementary Figures 5G, H**).

## 4 Discussion

We developed an interpretable model integrating static biomarker summaries with dynamic coupling features to predict response to anti-GD2 therapy combined with UCB-NK cell therapy. In this cohort of 42 patients, L2-regularized logistic regression yielded a cross-validated AUC of 0.873 and a Brier score of 0.131. These findings support further evaluation of serial data from routine peripheral blood testing as a complement to standard response assessment.

Among the fixed configurations evaluated, linear SVM had a slightly higher AUC, whereas L2-regularized logistic regression had numerically lower values for Brier score and Log loss. The latter was selected by balancing discrimination, the quality of probability estimates, and interpretability. This ranking is specific to the predictors and evaluation procedure used in this cohort and does not establish general superiority of linear models. Model differences may also reflect uncertainty arising from the small sample.

Laboratory-based models have been investigated for immunotherapy outcomes in NK/T-cell lymphoma and neuroblastic tumor staging. Differences in diseases, populations, treatments, prediction targets, and validation designs preclude direct performance comparisons. Our study combines static biomarker summaries with paired longitudinal coupling features to predict response to anti-GD2 therapy combined with UCB-NK cell therapy. The contribution is the evaluation of this integrated representation, rather than the development of a new classifier.

Exploratory survival analyses based on observed response showed better EFS among responders in the newly diagnosed high-risk cohort. However, the observed non-response group comprised only two patients. Kaplan-Meier estimates and log-rank comparisons may therefore be sensitive to individual event and censoring times. OS did not differ significantly between the observed response groups. OS captures mortality and may be influenced by subsequent treatment. In contrast, EFS includes events such as relapse or progression that may precede death, potentially allowing earlier assessment of disease control than OS. In the relapsed/refractory cohort, neither PFS nor OS differed significantly between groups defined by observed response. Clinical heterogeneity and differences in prior and subsequent treatments may contribute to these survival patterns. Survival differences were directionally similar between groups defined by predicted and observed response. However, small subgroups limit the precision and stability of these exploratory comparisons. Non-significant findings do not exclude an association between response status and survival.

The peripheral blood parameters included in our model may capture both the underlying disease biology and physiological changes associated with immunotherapy. SHAP analysis identified hemoglobin (HGB) as the most influential predictive feature. In the fitted model, higher HGB_mean contributed positively to predicted response. Low HGB has been associated with poorer prognosis in several cancer types (36, 37). In high-risk neuroblastoma, lower HGB levels are associated with an increased risk of acute hemorrhagic complications, which may complicate clinical management (38). Bone marrow is the most common metastatic site in neuroblastoma, and marrow infiltration is a strong predictor of poor outcome. Tumor cell infiltration into the bone marrow often impairs normal hematopoiesis, thereby affecting hematologic parameters and clinical presentation (39). These observations provide clinical context for the model finding, although the published outcomes differ from the treatment-response endpoint evaluated here. Therefore, the positive contribution of HGB_mean should be interpreted as a predictive association within this cohort. It does not establish that hemoglobin directly influences the efficacy of anti-GD2 therapy combined with UCB-NK cell immunotherapy.

Red blood cell distribution width–standard deviation (RDW-SD) reflects heterogeneity in erythrocyte size and may reflect systemic inflammation and bone marrow dysfunction. It has been associated with tumor progression and prognosis (40). Higher RDW-SD_baseline contributed positively to predicted response in this cohort. This finding appears inconsistent with reports that lower RDW during treatment and follow-up is associated with better prognosis in metastatic neuroblastoma (41). However, some studies have also found no significant association between RDW-SD and short-term mortality, long-term mortality, or most postoperative complications (42). Therefore, the positive contribution of RDW-SD_baseline should be interpreted as a cohort-specific model association rather than evidence of a beneficial biological effect.

Plateletcrit (PCT) reflects total circulating platelet volume. It has been associated with prognosis and treatment response in multiple malignancies (43–47). The role of basophils in tumor immunity is likely context-dependent, and both their abundance and activation state may change with the immunoregulatory microenvironment (48–50). The dynamic coupling features of PCT and BA% describe coordinated changes in these peripheral blood measurements. They may reflect shared disease- or treatment-related processes, but do not establish a biological interaction between platelets and basophils.

Serum amyloid A (SAA) is an acute-phase inflammatory protein synthesized predominantly in the liver in response to pro-inflammatory cytokines. SAA is typically elevated in infectious and inflammatory conditions and has been associated with poor prognosis in neuroblastoma (51). A study in hepatocellular carcinoma linked SAA to immunosuppression and resistance to PD-1 blockade (52). However, higher SAA_mean was associated with a greater predicted probability of response in the fitted model. This association does not establish that elevated SAA reflects beneficial treatment-induced immune activation. The biological basis of this association remains unclear and warrants investigation using paired longitudinal cytokine measurements and immune-cell profiling. Sodium (Na+) is essential for extracellular fluid balance and electrolyte homeostasis. In a murine neuroblastoma xenograft model, hyponatremia was associated with increased tumor growth and invasion-related molecular changes (53). These experimental findings do not establish how circulating sodium relates to response to anti-GD2 therapy combined with UCB-NK cell immunotherapy. The contributions of SAA_mean and Na+_mean should therefore be interpreted as predictive associations within this cohort, rather than evidence of specific mechanisms governing treatment response.

SHAP complemented permutation importance by quantifying signed feature contributions to individual predictions rather than changes in predictive performance. For this linear classifier, coefficient analysis already indicated association directions; SHAP added patient-level explanations.

This study has several limitations. First, the retrospective design may have introduced selection bias and residual confounding. Second, all patients were enrolled at a single center. Differences in patient management across institutions may therefore limit the model’s transportability. Third, the cohort was small (n = 42), resulting in imprecise performance estimates and an increased risk of overfitting. The cohort included only 13 non-responders, providing limited information for learning patterns associated with non-response. Selecting the final model from multiple candidates based on the same internal validation results may also yield optimistic estimates of its predictive performance. The small sample also limited the stability of the subgroup, survival, calibration, and model-explanation analyses. Although repeated stratified cross-validation reduced dependence on any single data partition, it could not fully address the uncertainty associated with the small cohort or substitute for external validation. No independent temporal or external cohort was available, and the model’s generalizability therefore remains unknown. Fourth, median imputation, standardization, and model fitting were performed within the training folds, reducing leakage during model estimation. However, exploratory feature screening and selection were partly informed by the full cohort before cross-validation. Information from held-out patients may therefore have indirectly influenced the final feature set, potentially producing optimistic performance estimates. Treatment regimens were also heterogeneous, which may limit treatment-specific interpretation of the identified predictors. Accordingly, the present findings should be regarded as exploratory. Future studies should incorporate the complete feature-selection and model-development pipeline within nested resampling, followed by prospective evaluation of a locked model in independent multicenter cohorts.

## Data Availability

The raw data supporting the conclusions of this article will be made available by the authors, without undue reservation.
