# Supplementary material for: An interpretable machine learning model for predicting response to anti-GD2 combined with umbilical cord blood-derived natural killer cell immunotherapy in neuroblastoma
Source: Front Immunol. 2026 Oct 29;17:1940534. doi: 10.3389/fimmu.2026.1940534 (PMC13754866; doi:10.3389/fimmu.2026.1940534)
Supplement: Supplementary file 1 [file DataSheet1.pdf]

# *Supplementary Material*

## 1 Supplementary Methods

### 1.1 Exploratory decision curve analysis

Exploratory DCA evaluated seven models in 42 patients for a hypothetical post-treatment non-response alert prompting additional clinical assessment. Non-response probabilities were calculated as 1 minus patient-level mean out-of-fold response probabilities from 50 repeats of stratified three-fold cross-validation.

Net benefit was compared with assessment of all or no patients across thresholds of 0.01–0.99 (0.01 increments). Paired net-benefit differences were defined as L2 minus linear SVM. Pointwise 95% percentile confidence intervals were estimated from 5000 paired patient-level bootstrap resamples, with fixed predictions and no model refitting. These intervals exclude full model-development uncertainty and are not simultaneous confidence bands.

## 2 Supplementary Results

### 2.1 Missing Data Summary

All 42 patients completed eight UCB-NK cell infusions, corresponding to 336 pre-infusion time points. Blood-test records were available for 322 time points; 14 (4.2%) were missing. Across all 336 time points, including those without records, unavailable measurements totaled 48 (14.3%) for PCT and 17 (5.1%) for RDW-SD. Corresponding counts were 14 (4.2%) for both BA% and HGB, 180 (53.6%) for Na+, and 192 (57.1%) for SAA.

Before imputation, the BA%–PCT coupling slope and concordance rate were both missing in the same three patients (3/42, 7.1%). HGB_mean, RDW-SD_baseline, Na+_mean, and SAA_mean were available for all 42 patients. Patient-level means were calculated from available non-missing measurements.

### 2.2 Model development

We began the stepwise feature expansion with BA%–PCT slope. This variable was identified in the preliminary within-patient dynamic screening because it captures the directional co-variation between BA% and PCT. Its inclusion as the initial feature does not imply that it showed superior performance across all univariable models. Rather, it was retained as a dynamic coupling feature for subsequent incremental evaluation. The order in which additional features were entered was determined on the basis of screening results, permutation importance, and clinical interpretability.

### 2.3 Exploratory decision curve analysis

Net-benefit rankings for L2-regularized logistic regression and linear SVM varied across thresholds. At 0.20, net benefit was 0.1786 for L2 and 0.1845 for SVM; at 0.50, the corresponding

values were 0.1429 and 0.1190. These thresholds were exploratory, not recommended clinical cutoffs.

Availability of all required measurements before the intended clinical decision was not verified. DCA therefore does not establish earlier detection of non-response, benefit from treatment changes, or improved clinical outcomes. The small cohort and non-nested feature and model selection may also yield optimistic net-benefit estimates.

## 2.4 Model explanation

SHAP waterfall plots provide local explanations for individual predictions. Red bars indicate positive contributions, and blue bars indicate negative contributions. RDW-SD_baseline contributed the largest positive effect (+2.62), offsetting negative contributions from other features (Supplementary Figure 5G). The patient shown in Supplementary Figure 5H was predicted as a non-responder.

# 3 Supplementary Figures and Tables

## 3.1 Supplementary Figures

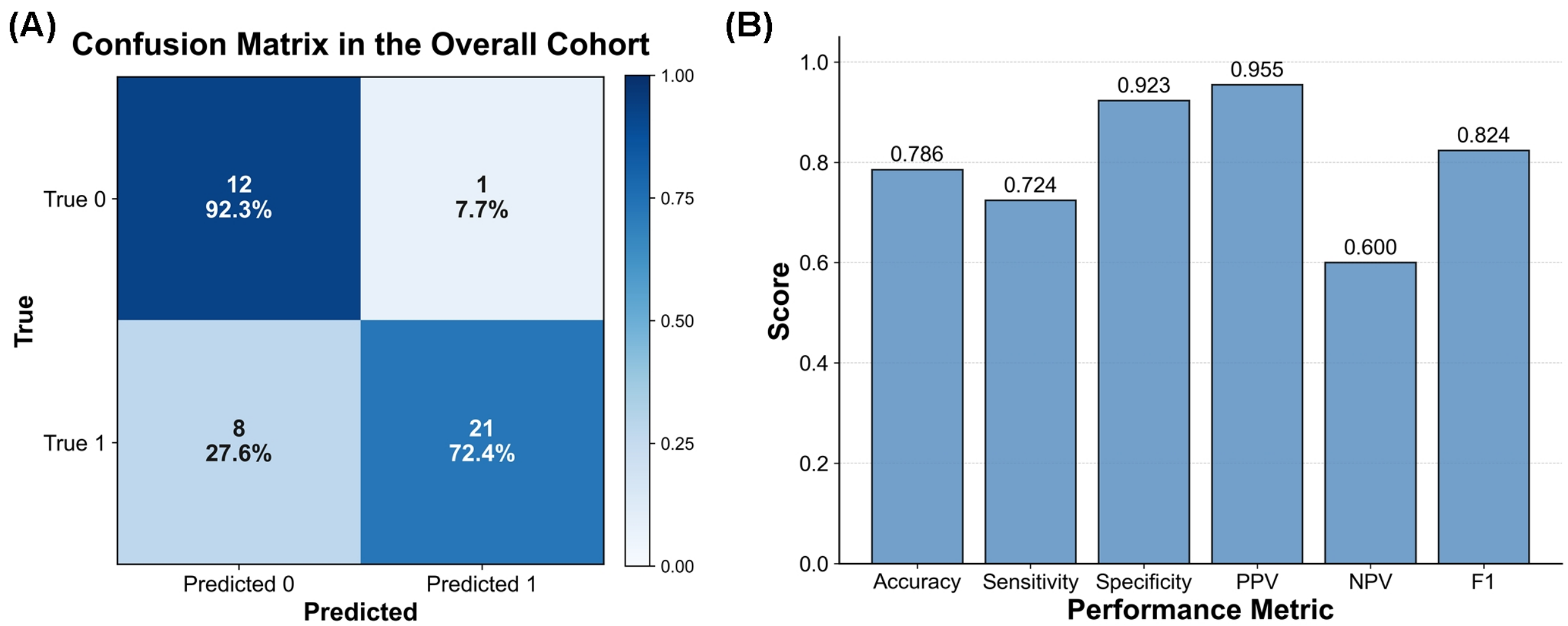


**Supplementary Figure 1.** Classification performance at the Youden threshold. (A) Confusion matrix obtained at the optimal threshold (0.740) using the Youden index. 0 indicates non-response; 1 indicates response. (B) Classification metrics at the Youden threshold.

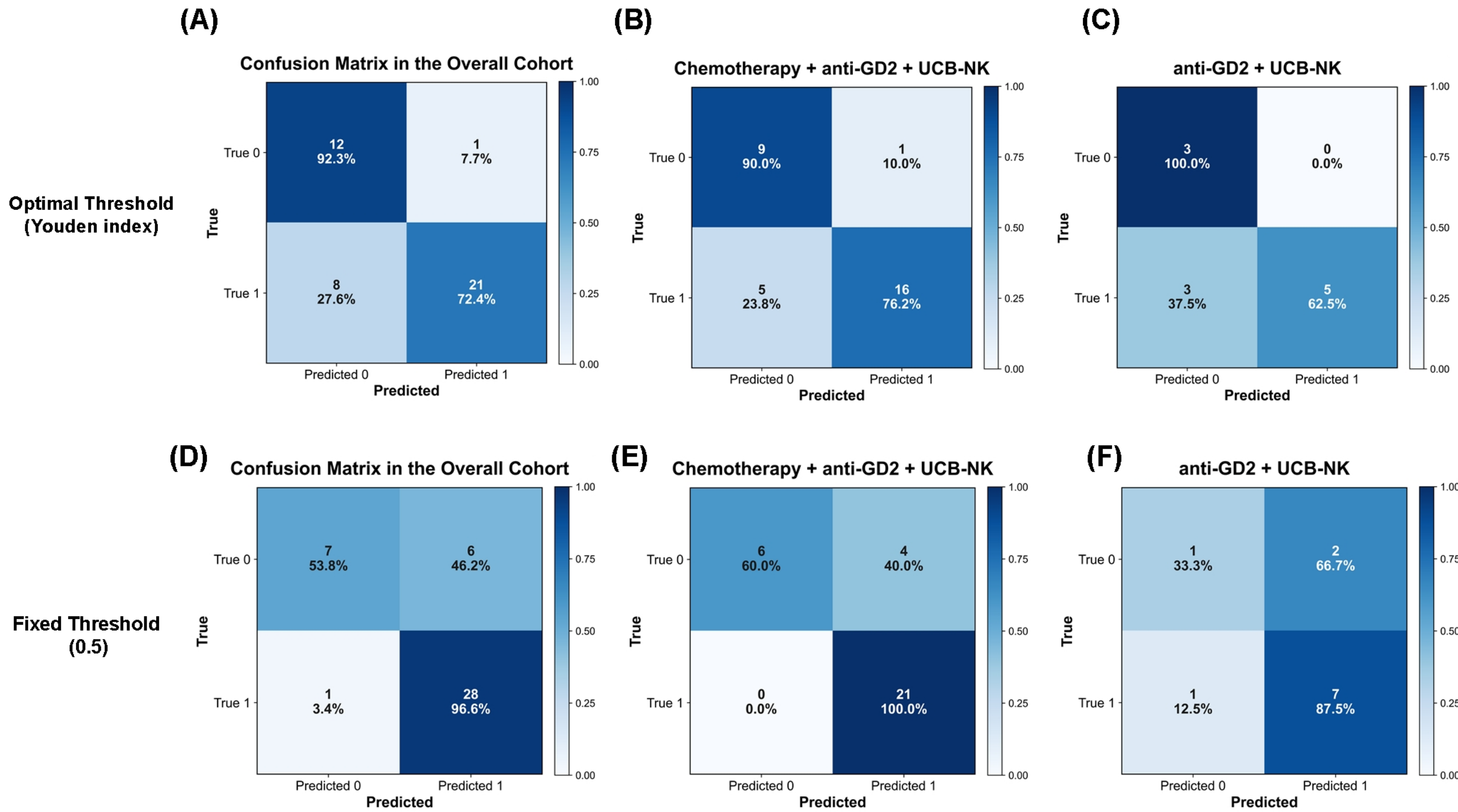


**Supplementary Figure 2.** Confusion matrices at different classification thresholds in the overall cohort and treatment subgroups. (A–C) Results using the Youden threshold. (D–F) Results using a fixed threshold of 0.5. (A, D) Overall cohort. (B, E) Chemotherapy combined with anti-GD2 therapy and UCB-NK cell immunotherapy. (C, F) Anti-GD2 therapy combined with UCB-NK cell immunotherapy without chemotherapy. Numbers in the matrices indicate sample counts.

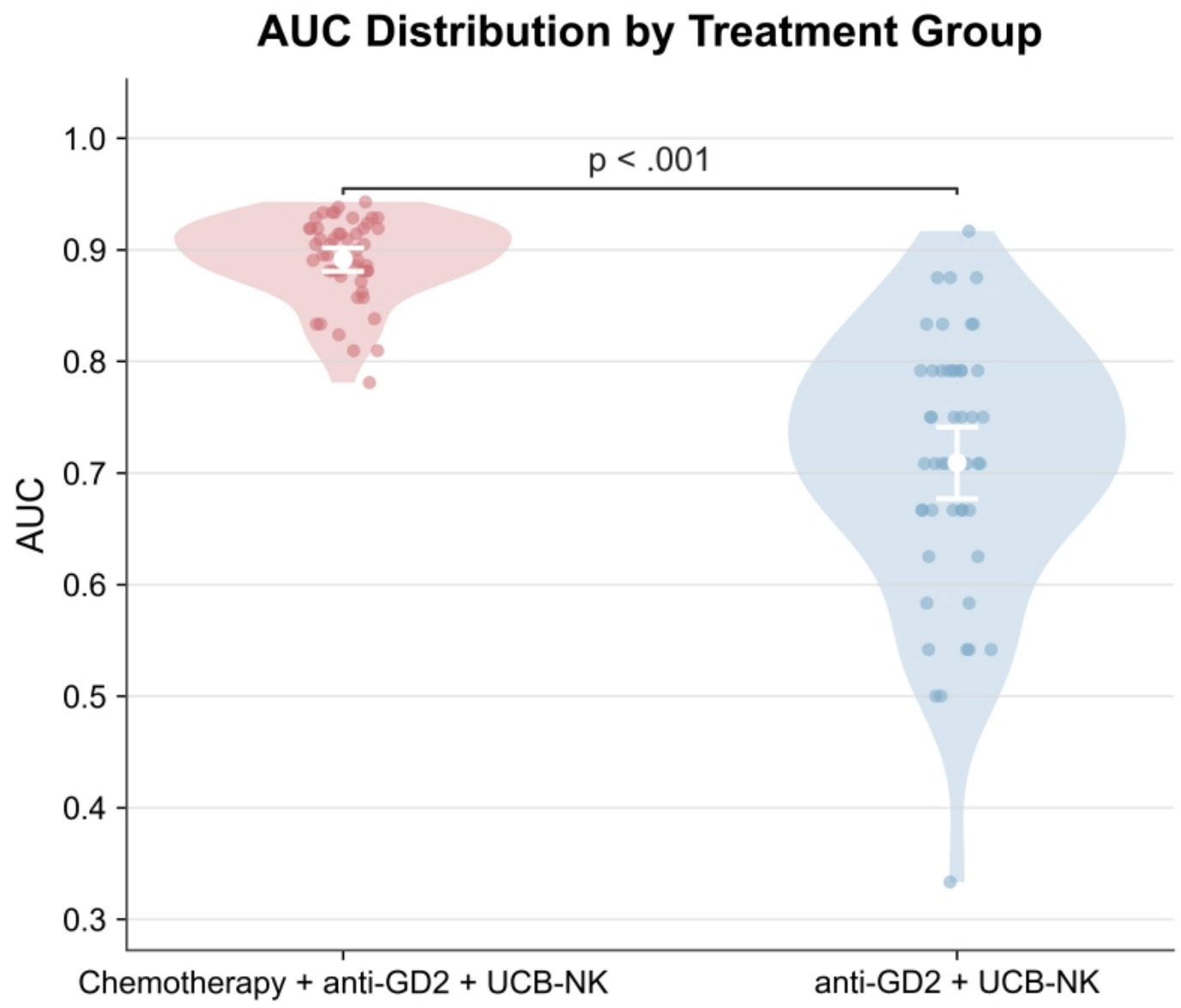


**Supplementary Figure 3.** AUC distributions across treatment subgroups obtained from repeated stratified cross-validation. Violin plots show the distribution of AUC values in each treatment subgroup. Individual points represent the subgroup-specific AUC estimate from each cross-validation repeat.

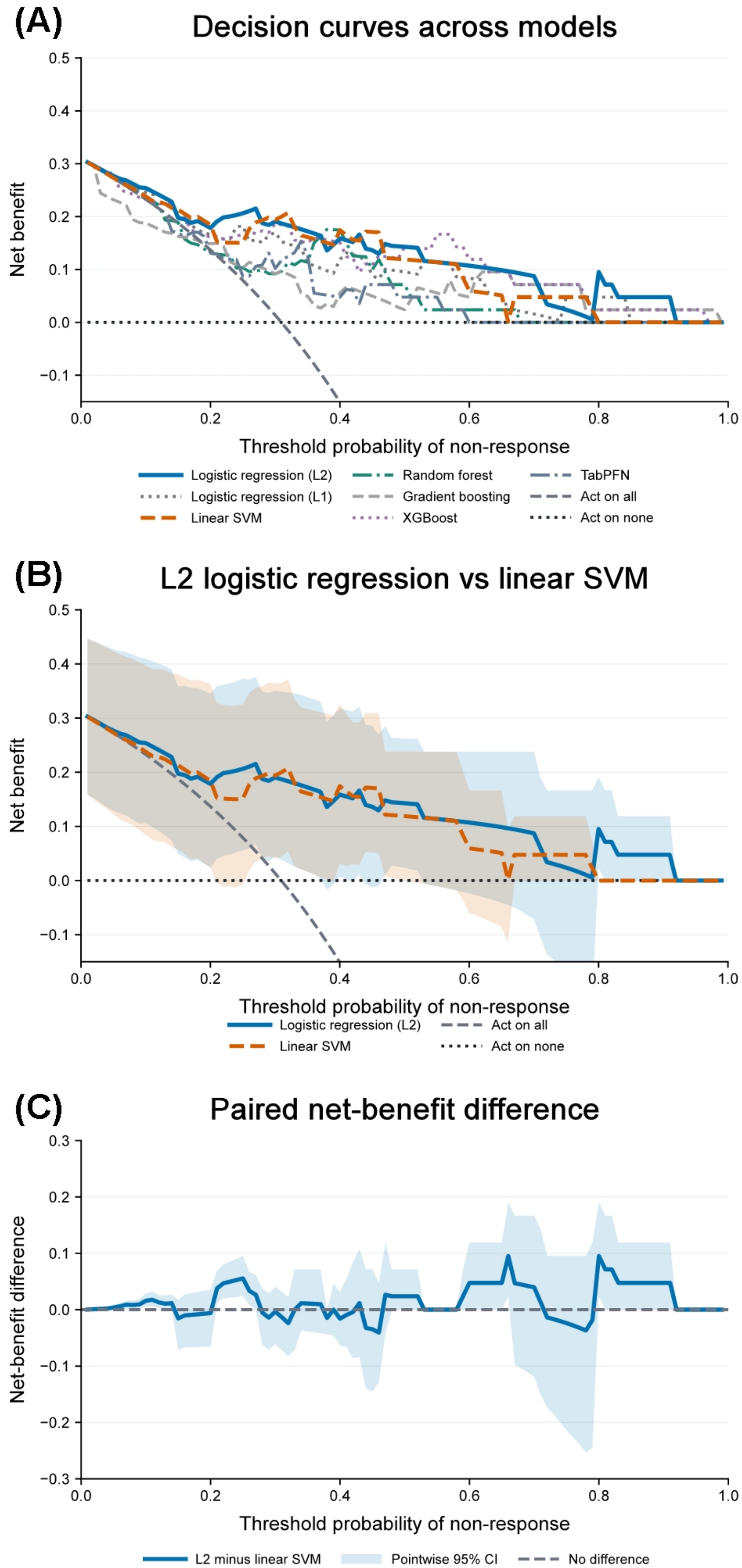
(A)
Decision curves across models
Net benefit
Threshold probability of non-response
Logistic regression (L2)
Logistic regression (L1)
Linear SVM
Random forest
Gradient boosting
XGBoost
TabPFN
Act on all
Act on none
(B)
L2 logistic regression vs linear SVM
Net benefit
Threshold probability of non-response
Logistic regression (L2)
Linear SVM
Act on all
Act on none
(C)
Paired net-benefit difference
Net-benefit difference
Threshold probability of non-response
L2 minus linear SVM
Pointwise 95% CI
No difference

**Supplementary Figure 4.** Exploratory decision curve analysis for non-response prediction. (A) Seven-model decision curves. (B) L2-regularized logistic regression and linear support vector machine (SVM). (C) Paired net-benefit difference (L2 minus SVM); positive values favor L2. Non-response probabilities were 1 minus each patient's mean out-of-fold response probability from stratified 3-fold cross-validation repeated 50 times. "Act on all" and "Act on none" assume hypothetical additional assessment for all or no patients, respectively. Thresholds (0.01-0.99) are exploratory, not clinically established. A and B display net benefit from -0.15 to 0.50, omitting values outside this range.

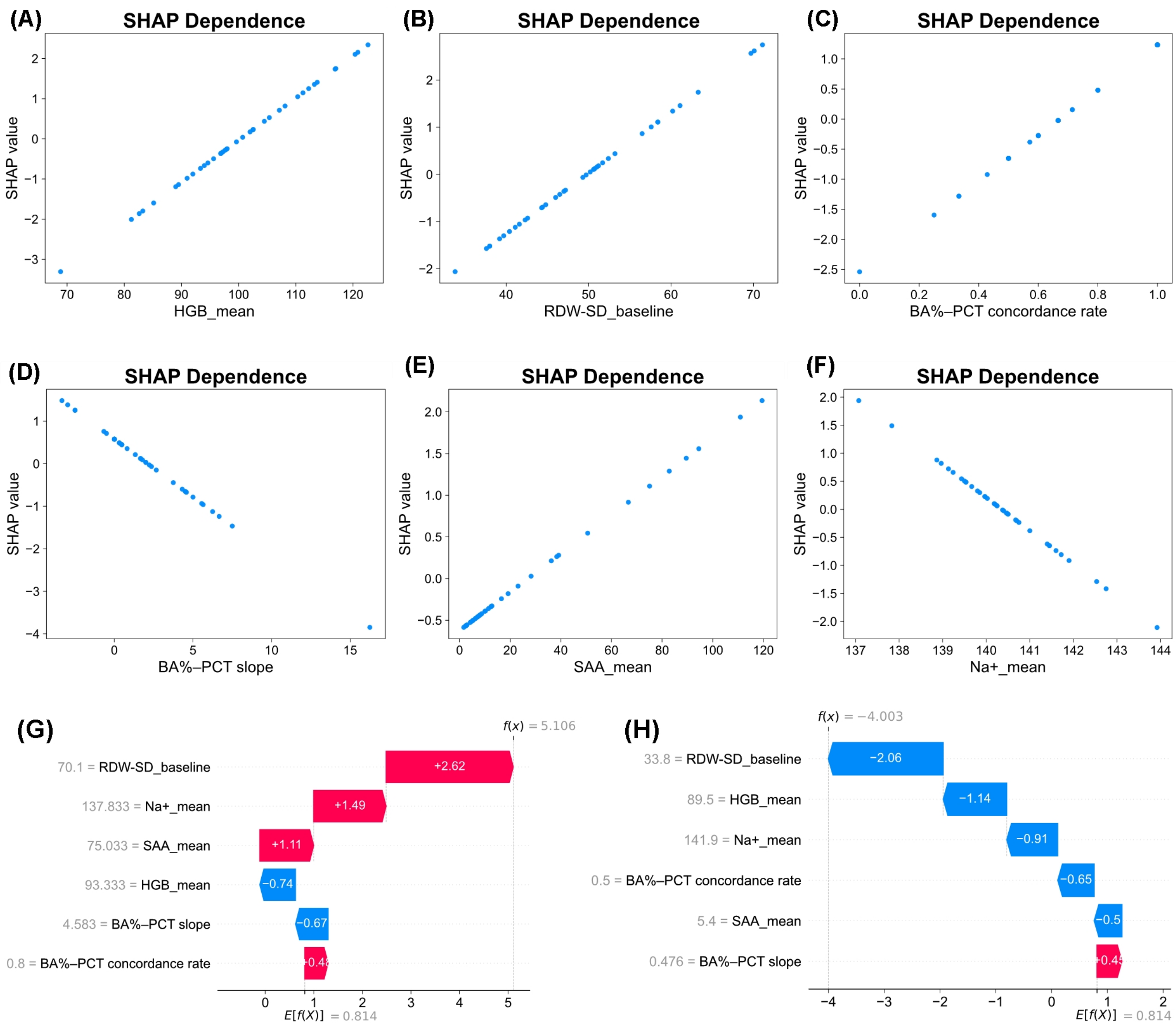


**Supplementary Figure 5.** SHAP dependence plots and waterfall plots. (A–F) Each plot shows how a single feature influences the model output. Individual points represent patients. The x-axis represents the original feature value, and the y-axis represents the SHAP value. (G, H) SHAP waterfall plots providing local explanations. (G) A patient predicted as a responder. (H) A patient predicted as a non-responder. Patients shown in the waterfall plots were selected using their out-of-fold predicted response probabilities.

### 3.2 Supplementary Tables

**Supplementary Table 1. Model Hyperparameter Settings**

| Model | Key hyperparameter settings |
|---|---|
| Logistic regression (L2) | penalty="l2"; C=1.0; solver="liblinear"; max_iter=5000; random_state=42 |
| Logistic regression (L1) | penalty="l1"; C=1.0; solver="liblinear"; max_iter=5000; random_state=42 |
| Linear SVM | C=1.0; loss="squared_hinge"; max_iter=10000; random_state=42; sigmoid probability calibration with three-fold internal cross-validation |
| Random forest | n_estimators=600; max_depth=None; min_samples_leaf=1; max_features="sqrt"; class_weight="balanced"; random_state=42 |
| Gradient boosting | n_estimators=100; learning_rate=0.1; max_depth=3; subsample=1.0; random_state=42 |
| XGBoost | n_estimators=300; learning_rate=0.05; max_depth=3; subsample=0.8; colsample_bytree=0.8; min_child_weight=1.0; gamma=0; reg_lambda=1.0; random_state=42 |
| TabPFN | tabpfn=8.0.3; pretrained TabPFN-3 classifier; n_estimators=8; softmax_temperature=0.9; balance_probabilities=False; device="auto"; random_state=0 |

Missing values were imputed using feature-specific medians estimated exclusively from each training fold. Standardization parameters were estimated within each training fold and applied only to logistic regression with L1 or L2 regularization and to the linear SVM. No external standardization was applied to the tree-based models or TabPFN. All models were evaluated on the same patient-level splits generated by stratified threefold cross-validation repeated 50 times. No data-driven hyperparameter tuning was performed during model comparison.

**Supplementary Table 2. Performance of stepwise feature expansion sets under repeated stratified cross-validation.**

| **Model** | **Features** | **AUC** | **SD** | ***p* value** |
|---|---|---|---|---|
| M0 | BA%–PCT slope | 0.614 | 0.045 | NA |
| M1 | BA%–PCT slope<br>HGB_mean | 0.669 | 0.054 | $p < .001$ |
| M2 | BA%–PCT slope<br>HGB_mean<br>RDW-SD_baseline | 0.742 | 0.044 | $p < .001$ |
| M3 | BA%–PCT slope<br>HGB_mean<br>RDW-SD_baseline<br>BA%–PCT concordance rate | 0.823 | 0.053 | $p < .001$ |
| M4 | BA%–PCT slope<br>HGB_mean<br>RDW-SD_baseline<br>BA%–PCT concordance rate<br>SAA_mean | 0.884 | 0.048 | $p < .001$ |
| M5 | BA%–PCT slope<br>HGB_mean<br>RDW-SD_baseline<br>BA%–PCT concordance rate<br>SAA_mean<br>CHE_slope | 0.886 | 0.049 | $p = .68$ |
| M6 | BA%–PCT slope<br>HGB_mean<br>RDW-SD_baseline<br>BA%–PCT concordance rate<br>CHE_slope | 0.827 | 0.058 | $p < .001$ |
| M7 | BA%–PCT slope<br>HGB_mean<br>RDW-SD_baseline<br>BA%–PCT concordance rate<br>Na+_mean | 0.869 | 0.050 | $p < .001$ |

**Supplementary Table 2. Performance of stepwise feature expansion sets under repeated stratified cross-validation. (continued)**

| Model | Features | AUC | SD | *p* value |
|---|---|---|---|---|
| M8 | BA%–PCT slope<br>HGB_mean<br>RDW-SD_baseline<br>BA%–PCT concordance rate<br>Na+_mean<br>SAA_mean | 0.915 | 0.041 | $p < .001$ |
| M9 | BA%–PCT slope<br>HGB_mean<br>RDW-SD_baseline<br>BA%–PCT concordance rate<br>Na+_mean<br>SAA_mean<br>CHE_slope | 0.919 | 0.034 | $p = .43$ |

M0–M9 denote candidate feature sets generated by stepwise expansion in a predefined order. AUC and SD represent the mean and standard deviation of AUC across repeated stratified cross-validation runs. AUC was calculated within each cross-validation repeat and then averaged across 50 repeats. These exploratory estimates are distinct from the primary comparison in 42 patients, which used imputation within training folds and calculated AUC from patient-level mean out-of-fold probabilities.

**Supplementary Table 3. Classification performance of the model in the overall cohort and across treatment subgroups at different classification thresholds.**

| Cohort | Overall | | Chemotherapy + anti-GD2 + UCB-NK | | anti-GD2 + UCB-NK | |
|---|---|---|---|---|---|---|
| Threshold | 0.500 | 0.740 | 0.500 | 0.740 | 0.500 | 0.740 |
| Accuracy | 0.833 (0.714-0.929) | 0.786 (0.643-0.905) | 0.871 (0.742-0.968) | 0.806 (0.677-0.935) | 0.727 (0.455-1.000) | 0.727 (0.455-0.909) |
| Sensitivity | 0.966 (0.885-1.000) | 0.724 (0.545-0.880) | 1.000 (1.000-1.000) | 0.762 (0.571-0.944) | 0.875 (0.600-1.000) | 0.625 (0.285-0.889) |
| Specificity | 0.538 (0.267-0.813) | 0.923 (0.750-1.000) | 0.600 (0.286-0.900) | 0.900 (0.667-1.000) | 0.333 (0.000-1.000) | 1.000 (1.000-1.000) |
| PPV | 0.824 (0.688-0.941) | 0.955 (0.850-1.000) | 0.840 (0.689-0.963) | 0.941 (0.800-1.000) | 0.778 (0.500-1.000) | 1.000 (1.000-1.000) |
| NPV | 0.875 (0.600-1.000) | 0.600 (0.381-0.824) | 1.000 (1.000-1.000) | 0.643 (0.364-0.909) | 0.500 (0.000-1.000) | 0.500 (0.000-0.857) |
| F1 | 0.889 (0.794-0.958) | 0.824 (0.683-0.923) | 0.913 (0.816-0.981) | 0.842 (0.700-0.950) | 0.824 (0.571-1.000) | 0.769 (0.443-0.941) |

The optimal threshold was determined using the Youden index, and performance was also evaluated at a fixed threshold of 0.5. Chemotherapy + anti-GD2 + UCB-NK indicates chemotherapy combined with anti-GD2 and UCB-NK cell therapy, and anti-GD2 + UCB-NK indicates anti-GD2 combined with UCB-NK cell therapy. Accuracy indicates the proportion of correctly classified samples overall. Sensitivity and specificity indicate the true-positive rate and true-negative rate, respectively. PPV and NPV reflect the probability that a positive or negative prediction is correct, respectively. F1 score is the harmonic mean of precision and recall. Confidence intervals were estimated using 2,000 non-stratified patient-level bootstrap resamples with replacement, with the indicated threshold held fixed in each resample.
